# Paraben Exposure in the General Taiwanese Population: Reference Values, Personal Care Products, and Cumulative Risk Assessment

**DOI:** 10.1002/kjm2.70208

**Published:** 2026-04-11

**Authors:** Jung‐Wei Chang, Yen‐Hsuan Huang, Hsin‐Chang Chen, Vinoth Kumar Ponnusamy, Wan‐Ting Chang, Po‐Chin Huang

**Affiliations:** ^1^ Institute of Environmental and Occupational Health Sciences, School of Medicine National Yang Ming Chiao Tung University Taipei Taiwan; ^2^ Department of Applied Chemistry, College of Science and Engineering National Chiayi University Chiayi City Taiwan; ^3^ Research Center for Precision Environmental Medicine Kaohsiung Medical University Kaohsiung City Taiwan; ^4^ Department of Medicinal and Applied Chemistry Kaohsiung Medical University Kaohsiung City Taiwan; ^5^ Ph.D. Program in Environmental and Occupational Medicine, College of Medicine Kaohsiung Medical University Kaohsiung City Taiwan; ^6^ National Institute of Environmental Health Sciences National Health Research Institutes Miaoli Taiwan; ^7^ Department of Medical Research, China Medical University Hospital China Medical University Taichung Taiwan

**Keywords:** parabens, reference values, Taiwanese general population

## Abstract

Parabens (parahydroxybenzoates), which are frequently used as preservatives in pharmaceuticals, foodstuffs, and personal care products, have recently attracted considerable attention due to their adverse health effects and endocrine‐disrupting properties. This study aimed to determine the background urinary concentrations of parabens in a representative sample of the general population in Taiwan. A total of 1967 participants were enrolled in this cross‐sectional study. The subjects were recruited from the Taiwan Environmental Survey for Toxicants (2013–2016). Urine samples were collected from participants, and questionnaires were administered. Urinary concentrations of parabens, including methylparaben (MeP), ethylparaben (EtP), propylparaben (PrP), and butylparaben (BuP), were quantified through ultra‐high‐performance liquid chromatography coupled with tandem mass spectrometry. Overall, 95th percentile reference values (RV95s) were used to establish the background concentrations of these parabens. The highest geometric mean concentration was obtained for MeP (357 μg/L), followed by PrP (89.3 μg/L), EtP (34.2 μg/L), and BuP (5.31 μg/L). Urinary concentrations of these parabens were higher in adults than in minors. The RV95s of paraben were 954, 101, 219, and 14.2 μg/L for MeP, EtP, PrP, and BuP, respectively. Of the 1967 participants, 55.4% had a HI value that exceeded the threshold of 1. This finding indicates a potential concern under conservative screening assumptions, rather than definitive evidence of adverse health risk. PrP was identified as the primary determinant of HI, accounting for 71.4% of the observed effect. The RV95s for the urinary concentrations of particular parabens are significantly higher in Taiwan than in other countries. Moreover, a notable correlation was observed between personal care product use and the urinary paraben concentrations. Further evaluation of the health implications of paraben exposure among the Taiwanese population is warranted.

## 1 Introduction

Across a wide range of pH values and temperatures, parabens are stable and water soluble and are used as antimicrobial preservatives. Due to the high demand for products with extended shelf lives, the use of parabens as preservatives has increased. Parabens are employed as preservatives in personal care products, pharmaceuticals, and food, and they may have long‐term environmental effects [1, 2]. Because of their low toxicity, broad inertness, and affordability, the use of parabens is widely used in daily life products, particularly pharmaceuticals and personal care products [3, 4]. The most commonly used parabens are methylparaben (MeP), ethylparaben (EtP), propylparaben (PrP), and butylparaben (BuP). Exposure to parabens has increased due to their widespread use in everyday products, especially personal care products. Parabens are also used in food and cosmetics.

Notably, in vitro and in vivo studies have demonstrated that parabens are endocrine disruptors that are capable of interfering with the functions of a range of hormones, including androgens, estrogens, progesterone, and glucocorticoids, and peroxisome proliferator–activated receptors [3]. Furthermore, parabens have been shown to exert adverse effects on the reproductive system of rats [5, 6].

Reference values (RVs) for exposure to certain substances are typically based on the 95th percentile of the pollutant concentration in the study population (i.e., 95th percentile reference value [RV95]) and its 95% confidence interval (CI). Human biomonitoring studies tend to use RV95 as the benchmark RV. Exposure concentrations exceeding the RV95 suggest that individuals may require greater attention regarding exposure risk.

While many countries have restricted or prohibited the use of certain parabens in cosmetics since the 2000s, Taiwan does not implement a universal ban. Instead, the Taiwan Food and Drug Administration (TFDA) imposes specific limits on long‐chain parabens such as propylparaben (PrP) and butylparaben (BuP), requiring warning labels for products intended for use by young children. Short‐chain parabens, including methylparaben (MeP) and ethylparaben (EtP), are permitted within defined concentration limits. The total concentration of PrP and BuP (and their salts) is generally restricted to a maximum of 0.14% in finished cosmetic products, consistent with international standards, including EU and SCCS recommendations. Other long‐chain or branched parabens—such as isopropylparaben, isobutylparaben, phenylparaben, benzylparaben, and pentylparaben—are prohibited in cosmetics in Taiwan in alignment with ASEAN and EU regulations. In general, most of the Taiwanese population is exposed to parabens [7]. Participants who used more personal care products were more likely to have higher concentrations of PrP (above the 75th percentile) [adjusted odds ratio (aOR): 1.79, 95% CI: 1.01–3.15] and BuP [aOR: 1.78, 95% CI: 1.03–3.07]. The study revealed that the majority of Taiwanese individuals have elevated levels of four distinct types of parabens in their bodies, particularly MeP and PrP. The level of these four parabens was higher than those reported in other countries. The median and 95th percentile hazard index (HI) (the sum of the hazard quotients [HQs] of each paraben) was found to be 1.10 and 4.39‐fold higher than the acceptable cumulative threshold (HI < 1), with PrP accounting for 90% of the HI.

Accordingly, the routine surveillance and ongoing biomonitoring of parabens are necessary. Compared with the limited scale of our previous study, it is of paramount importance to establish a RV for urinary parabens in a representative sample of the Taiwanese population [7]. The derivation of RVs for the general population followed the guidelines of the International Federation of Clinical Chemistry (IFCC) and the International Union of Pure and Applied Chemistry (IUPAC), as previously detailed in our report [8, 9]. Establishing these benchmarks facilitates comparisons of paraben exposure levels in individuals and the general population and helps define background levels. This approach enables the identification of exposure levels that may be of concern. The RV95 can also be used to monitor temporal trends in environmental paraben levels, thereby evaluating the effectiveness of pollution‐reduction policies. By understanding current background levels and identifying areas requiring attention, policymakers can develop targeted interventions and regulations.

In this study, we assessed the urinary concentrations of selected parabens by using Taiwan Environmental Survey for Toxicants (TEST) data from the 2013–2016 national survey to establish background exposure levels for the general Taiwanese population. Additionally, we examined age, gender, and region‐based variations in exposure concentrations.

## 2 Materials and Methods

### 2.1 Study Population Collection and Ethical Approval

This study used data from the 2013–2016 Taiwan Environmental Survey for Toxicants (TEST). Participant recruitment was conducted of the TEST, which was designed to align with the sampling framework of the National Nutrition and Health Survey in Taiwan (NAHSIT) to enhance population representativeness. Taiwan was stratified into five major geographical regions (northern, central, southern, eastern, and offshore islands), comprising 22 cities and counties. Townships within each city or county were classified according to population density and degree of urbanization. Using this stratification scheme, NAHSIT randomly selected representative townships, from which TEST recruited participants across all age groups. A total of 1967 participants aged 7–97 years were included in the analysis. Eligibility criteria excluded pregnant or breastfeeding women, individuals with severe diseases (e.g., cancer), hospitalized or incarcerated individuals, and non‐Taiwanese residents. Ultimately, 17 townships from 11 cities and counties across four main regions and the offshore Penghu County were included [7].

Individuals with insufficient urine or blood samples or those unable to provide samples for analysis were also excluded.

Sampling was conducted between 2013 and 2016. Participants were recruited during health checkup events held at elementary schools and community centers. During the health checkups, first morning urine samples and fasted morning blood samples were collected, and a questionnaire was administered to obtain basic demographic and exposure information.

The study was conducted in accordance with the Declaration of Helsinki, and the protocol was approved by the Research Ethics Committee of National Health Research Institutes and National Yang Ming Chiao Tung University (No. EC1020206 and No. YM110074E) of Taiwan. All donors involved in this study signed an informed consent form before the sample collection.

### 2.2 Paraben Analysis

The analytical methodology used in this study has been described in detail elsewhere [7, 10]. In brief, first morning urine samples were collected from participants after more than 8 h of fasting. Samples were stored in precleaned amber glass bottles washed with acetonitrile and stored at −20°C until analysis.

Quantification of four urinary parabens was performed using isotope dilution‐ultra‐performance liquid chromatography tandem mass spectrometry (ID‐UPLC‐MS/MS), as previously described [10]. In brief, 100 μL of urine was spiked with 20 μL of methanol containing four stable‐isotope‐labeled internal standards (^13^C_6_‐MeP, ^13^C_6_‐EtP, PrP‐d_7_, and BuP‐d_9_) in a 1.5 mL microcentrifuge tube. Enzymatic hydrolysis was carried out by adding 5 μL of β‐glucuronidase (≥ 85,000 U/mL, *Helix pomatia* ) and 20 μL of 1.0 M ammonium acetate, followed by incubation at 40°C for 1 h. The reaction was quenched with 135 μL of 0.1% aqueous formic acid, yielding a final volume of 280 μL. The mixture was then loaded onto a supported liquid extraction (SLE) cartridge. Analytes were eluted twice with 0.9 mL of dichloromethane, and the combined eluate was evaporated to dryness at 30°C. The residue was reconstituted in 200 μL of 50% aqueous methanol for UPLC‐MS/MS analysis.

A Waters ACQUITY UPLC System with a Thermo Scientific Hypersil GOLD column was used for chromatographic separation and quantification. Measurements were conducted following the same methodology as that used in the TEST [10]. The supported liquid extraction technique provides an acceptable recovery rate for analytes and minimizes interferences in liquid chromatography–mass spectrometry bioanalysis [10]. The current experimental results demonstrated that the recovery rates of parabens in artificial urine were within acceptable ranges and were stable. The mean recovery rates (with relative standard deviations in brackets) for the parabens at low, medium, and high concentrations were 91.6%–100.9% (5.4%–10.5%), 84.4%–99.5% (1.9%–7.1%), and 86.8%–98.4% (1.7%–13.7%), respectively, all meeting the European Medicines Agency standards. The matrix effects of the three analytes in artificial urine ranged from 80.3% to 96.7%, with precision below 14.2%. The results presented in this study met the European Medicines Agency standards.

The intra‐ and inter‐batch accuracy (expressed as the mean spiked recovery) and precision (expressed as the relative standard deviation) also met the validation criteria specified by the European Medicines Agency standards (EMA). For the low, medium, and high concentrations, the intra‐batch accuracy (precision) ranged from 100.8% to 111.6% (precision ≤ 12.6%), 90.0% to 112.3% (precision ≤ 10.1%), and 87.7% to 103.1% (precision ≤ 13.3%), respectively. Inter‐batch accuracy (precision) ranged from 97.8% to 103.4% (precision ≤ 6.3%), 95.5% to 101.0% (precision ≤ 7.4%), and 97.5% to 105.3% (precision ≤ 7.8%), respectively.

Prior to the analysis of the 20 actual urine samples per batch, quality control assessments were conducted using 47 batches of quality control samples to evaluate precision and accuracy. The accuracy (precision) at low, medium, and high concentrations ranged from 87.63%–112.85% (precision ≤ 14.67%), 87%–112.48% (precision ≤ 14.95%), and 87.23%–112.98% (precision ≤ 13.38%), respectively.

### 2.3 Risk Assessment

We calculated the daily intake (DI) of the four parabens and stratified the results into five age groups: children (aged ≥ 7 to < 12 years), adolescents (aged ≥ 12 to < 18 years), young adults (aged ≥ 18 to < 40 years), middle‐aged adults (aged ≥ 40 to < 65 years), and older adults (aged ≥ 65 years). DI was estimated using a back‐calculation approach based on urinary paraben concentrations [7, 11].

The DI is a comprehensive metric that estimates the total oral‐equivalent potential intake via three routes (oral, dermal, and inhalation) and all possible exposure sources (e.g., food preservatives or PPCPs usage). The equation used to estimate DI from urinary concentrations, similar to that applied for phthalates, has been widely used and validated for parabens in human biomonitoring studies [12, 13]. This approach involves measuring parent parabens in urine (MeP, EtP, PrP, BuP) and applying compound‐specific excretion fractions and metabolism‐based conversion factors to estimate external intake, which is subsequently compared to acceptable daily intake (ADI) values for risk assessment. The DI of each paraben is calculated using the following equation [14, 15]:

(1) $$\text{Daily intake}\left(\mathrm{\mu g}/\mathrm{kg}/\mathrm{day}\right)=\frac{\mathrm{UE}\times CE_{\text{smoothed}}}{F_{\mathrm{UE}}\times \mathrm{BW}\times 1000}\times \mathrm{MW}$$

where UE is the urinary excretion concentration of a paraben, which is expressed as μmol/g creatinine. The smoothed creatinine excretion rate (CE_smoothed_) represents the daily creatinine excretion rate [7]. We have described the formulae for estimating CE_smoothed_ elsewhere [11].

For adults (age ≥ 18 years):

$$\mathrm{CE}=1.93\times \left(140-\mathrm{age}\right)\times \text{body weigh}t^{1.5}\times \text{heigh}t^{0.5}\times 10^{-3}\left(\text{for male}\right)$$

$$\mathrm{CE}=1.64\times \left(140-\mathrm{age}\right)\times \text{body weigh}t^{1.5}\times \text{heigh}t^{0.5}\times 10^{-3}\left(\text{for female}\right)$$

For minors (age ≥ 7 to < 18 years):

$$\begin{array}{c} \mathrm{CE}=\text{height}\times \left\{6.265+0.0564\times \left(\text{height}-168\right)\right\}\\ \;\left(\text{for boys with}\;a\;\text{height of}<168\;\mathrm{cm}\right) \end{array}$$

$$\begin{array}{c} \mathrm{CE}=\text{height}\times \left\{6.265+0.2550\times \left(\text{height}-168\right)\right\}\\ \;\left(\text{for boys with}\;a\;\text{height of}\geq 168\;\mathrm{cm}\right)\\ \;\mathrm{CE}=2.045\times \text{height}\times \exp\;\left\{0.01552\times \left(\text{height}-90\right)\right\}\;\left(\text{for girls}\right) \end{array}$$

*F*_UE_ is the urinary excretion rate of each paraben (MeP: 17.4%, EtP: 13.7%, PrP: 8.6%, and BuP: 5.6%). MW is the molecular weight of a paraben [15]. An examination was conducted on the non‐cancerous risks posed by parabens. In practical applications of the HI approach, regulatory health‐based guidance value (HBGV) (e.g., the ADI, tolerable daily intake [TDI] or reference dose [RfD]) has been utilized as the denominator in HQ. The HI formula was as follows:

(2) $$\mathrm{HI}=HQ_{\mathrm{MeP}}+HQ_{\mathrm{EtP}}+HQ_{\mathrm{PrP}}+HQ_{\mathrm{BuP}}$$

the HQ formula was as follows:

The HQ was employed to assess the risks associated with a specific paraben, and the HI was employed to evaluate the overall risk posed by multiple parabens (MeP + EtP + PrP + BuP). HQ and HI scores of > 1 indicated health risks. The calculations are described in detail elsewhere. HQ scores are best calculated using regulatory health‐based guidance values. The relevant formula is as follows:

(3) $$\mathrm{HQ}=\frac{\mathrm{DI}}{\text{HBGV}}$$

The calculation of health‐based guidance values entails the division of the point of departure, which may be either the No Observed Adverse Effect Level or No Observed Effect Level, by the corresponding uncertainty factors.

The health‐based guidance value selected for the sum of MeP and EtP was an acceptable DI of 10 mg/kg/day [16, 17]. The health‐based guidance value of 0.02 mg/kg/day was employed in the HQ/HI approach [15]. However, there was no official health‐based threshold value for PrP and BuP. Therefore, we used a conservative no‐observable effect level (NOEL) of 2 mg/kg bw/day [15, 18], based upon reproductive toxicity studies in rodents [19]. The point of departure (e.g., NOEL) and an uncertainty factor (UF) of 100 were combined to obtain HBGV (0.02 mg/kg bw/day) for the HQ/HI approach [15].

### 2.4 Statistical Analysis

Descriptive statistics were used to summarize participant demographic characteristics, including (gender, age, body mass index, region, marital status, educational level, and household income), environmental exposures (exposure to secondhand smoke and pesticides), and lifestyle habits (smoking, alcohol/tea/coffee consumption, betel nut chewing, incense burning, use of personal care products, diet, and medication habits). For urinary paraben concentrations, the detection rate, geometric mean, minimum value, maximum value, and selected percentiles (25th, 50th, 75th, and 95th percentiles) were calculated. Additionally, the bootstrap method was used to estimate the 95% CIs for these parameters.

As paraben concentrations were not normally distributed, nonparametric tests were applied. The Mann–Whitney *U* test was used to assess differences between two groups and the Kruskal–Wallis test was employed to evaluate differences across multiple groups (e.g., by sex, age group, and region).

## 3 Results

### 3.1 Participant Demographic Variables

Data was obtained from the 2013, 2015, and 2016 TEST surveys. In total, 1967 participants were recruited including 1345 adults and 622 minors. The gender ratio was approximately 1:1 for both adults and minors.

In total, 43.9% of the adult participants were aged 40–64 years. Moreover, 55% of the minors were aged 7–11 years (Table 1). Average body mass index values were 24.3 kg/m^2^ for adults and 19.1 kg/m^2^ for minors. In terms of the residential area, most of the adults lived in northern Taiwan (35.1%), followed by southern Taiwan (27.3%), central Taiwan (20.3%), eastern Taiwan (11.2%), and remote islands (6.1%). A similar distribution was found for minors. These distributions aligned with the general population structure in Taiwan as reported by the Taiwanese government.

**TABLE 1:** Characteristics of the study population (*N* = 1967).

Variables	Adults (≧ 18 years, *N* = 1345)		Children/adolescents (< 18 years, *N* = 622)	
	*N* (%)	Mean ± SD	*N* (%)	Mean ± SD
Gender				
Male	655 (48.7)		309 (49.7)	
Female	690 (51.3)		313 (50.3)	
Age (years, mean ± SD)	1345	52.4 ± 17.6	622	11.9 ± 3.0
18–39/7–11	370 (27.5)		342 (55.0)	
40–64/12–17	591 (43.9)		280 (45.0)	
65 and older	384 (28.6)		0 (0.0)	
BMI (kg/m^2^, mean ± SD)	1345	24.3 ± 4.1	622	19.1 ± 4.4
Region				
Northern Taiwan	472 (35.1)		202 (32.5)	
Central Taiwan	273 (20.3)		156 (25.1)	
Southern Taiwan	367 (27.3)		155 (24.9)	
Eastern Taiwan	150 (11.2)		76 (12.2)	
Remote islands	83 (6.1)		33 (5.3)	
Marital status				
Single	266 (19.8)		619 (99.5)	
Married	1031 (76.6)		3 (0.5)	
Divorce/widowed	48 (3.6)		0 (0)	
Education				
≦ Elementary school	267 (19.9)		359 (57.7)	
Junior high school	152 (11.3)		164 (26.4)	
Senior high school	431 (32.0)		96 (15.4)	
≧ College/graduates	495 (36.8)		3 (0.5)	
Annual family income^a^				
< 15,625	588 (44.7)		235 (39.0)	
15,625~31,250	449 (34.1)		224 (37.1)	
> 31,250	279 (21.2)		144 (23.9)	
Cigarette smoking^b^				
Yes	249 (18.5)		5 (0.8)	
No	1095 (81.5)		617 (99.2)	
Alcohol consumption^c^				
Yes	71 (5.5)		1 (0.2)	
No	1212 (94.5)		620 (99.8)	
Tea drinking^d^				
Yes	725 (53.9)		294 (47.3)	
No	619 (46.1)		328 (52.7)	
Coffee drinking^d^				
Yes	614 (45.7)		46 (7.4)	
No	731 (54.3)		576 (92.6)	
Betel nut chewing^e^				
Yes	39 (2.9)		1 (0.2)	
No	1304 (97.1)		621 (99.8)	
PCPs usage^f^				
Yes	864 (68.1)		490 (80.2)	
No	404 (31.9)		121 (19.8)	

^a^ The currency exchange rate of converting USD to new Taiwan dollar is 1:32.

^b^ Subjects who self‐reported consuming at least one cigarette per day.

^c^ Subject who self‐reported consuming at least one bottle of alcohol drink per week.

^d^ Subjects who self‐reported consuming at least one cup of tea or coffee per week.

^e^ Subject who self‐reported chewing at least one betel nut per week.

^f^ Use of one or more personal care products.

Among adult participants, 76.6% were married, 19.8% were single, and 3.6% were divorced or widowed. Regarding the educational level, 36.8% of the adults had a college or higher degree. Regarding the household income, 44.7% of the adults had an annual household income of < NT$15,625.

In terms of lifestyle habits, adults were more likely than minors to smoke, drink alcohol, tea, or coffee, or chew betel nut. Moreover, 81.5% of the adult participants were nonsmokers, and 94.5% did not have the habit of drinking alcohol. Additionally, 68.1% of the adult and 80.2% of the minor participants reported using personal care products.

### 3.2 Distribution of Urinary Paraben Concentration by Sex

Distributions of paraben concentrations were determined by calculating the detection rates, maximum and minimum values, interquartile ranges (IQRs), and geometric means (Table 2). The detection rate for all parabens was 100% among adults. Among minors, the detection rates for the four parabens were as follows: MeP (100%), EtP (95.0%), PrP (96.6%), and BuP (99.0%).

**TABLE 2:** Urinary levels of parabens in Taiwanese with different sex and age.

Parabens	*N*	% > LOD	Min	GM (95% CI)	Selected percentiles				Max	*p*
(μg/L)					25th (95% CI)	50th (95% CI)	75th (95% CI)	95th (95% CI)		
MeP										
Total population	1967	100	0.42	357 (344–372)	257 (248–266)	404 (385–418)	603 (581–626)	954 (921–984)	1211	
Males	964	100	0.42	356 (335–376)	257 (245–267)	405 (383–429)	611 (588–654)	971 (918–990)	1210	0.366^a^
Females	1003	100	0.55	359 (341–377)	259 (242–273)	401 (376–418)	594 (552–626)	929 (906–976)	1211	
7–17 years	622	100	0.42	288 (261–315)	234 (206–251)	378 (351–406)	565 (542–619)	911 (862–956)	1110	< 0.001***^b^
7–11	342	100	0.42	275 (241–311)	218 (196–242)	364 (323–398)	555 (522–620)	892 (829–962)	1076	0.157^c^
12–17	280	100	0.47	304 (259–350)	249 (219–264)	401 (364–432)	579 (541–660)	918 (875–995)	1110	
≧ 18 years	1345	100	54.2	395 (383–408)	271 (257–283)	414 (396–430)	612 (589–644)	971 (938–991)	1211	
18–39	370	100	54.2	360 (336–381)	233 (219–256)	358 (334–415)	589 (539–649)	933 (870–991)	1166	0.004**^c^
40–64	591	100	58.6	407 (388–426)	282 (263–302)	422 (399–448)	613 (574–650)	961 (929–1018)	1205	
≧ 65	384	100	65.0	425 (392–440)	281 (263–306)	428 (400 –460)	646 (602–701)	986 (936–1061)	1211	< 0.001***^d^
EtP										
Total population	1967	98.4	0.05	34.2 (32.4–35.8)	24.4 (23.6–25.9)	40.5 (39.0–42.2)	62.5 (60.3–64.8)	101 (98.6–105)	134	
Males	964	99.1	0.05	33.6 (31.2–36.1)	24.2 (22.7–25.9)	41.2 (38.8–43.6)	64.5 (62.0–67.8)	102 (98.5–108)	130	0.521^a^
Females	1003	99.3	0.05	34.7 (32.4–36.8)	24.6 (23.6–26.1)	40.0 (37.8–42.1)	60.2 (57.2–63.4)	101 (94.2–106)	134	
7–17 years	622	95.0	0.05	25.0 (21.7–28.2)	21.4 (19.5–23.6)	38.0 (34.9–41.1)	60.2 (56.5–64.5)	101 (93.5–104)	134	0.001**^b^
7–11	342	94.7	0.05	23.9 (19.8–28.4)	20.9 (16.9–24.6)	38.2 (34.2–42.5)	59.7 (54.9–65.4)	100 (88.2–105)	122	0.838^c^
12–17	280	95.4	0.05	26.3 (21.6–31.5)	22.3 (19.5–24.2)	37.8 (32.9–42.0)	62.2 (55.7–66.7)	101 (92.5–107)	134	
≧ 18 years	1345	100	4.34	39.5 (38.8–40.8)	25.5 (24.4–26.8)	42.0 (39.5–43.6)	63.4 (60.5–66.7)	103 (98.7–107)	130	
18–39	370	100	5.71	40.2 (37.8–42.5)	26.8 (24.5–28.4)	42.9 (39.5–45.2)	62.6 (58.9–67.3)	98.5 (93.3–107)	130	0.239^c^
40–64	591	100	4.34	40.4 (38.5–42.5)	25.4 (24.1–27.2)	42.9 (38.7–45.6)	65.7 (61.8–72.0)	109 (101–112)	130	
≧ 65	384	100	5.92	37.5 (35.1–39.9)	24.2 (22.2–26.8)	39.4 (35.8–43.2)	59.6 (55.9–67.2)	98.8 (92.8–106)	128	< 0.001***^d^
PrP										
Total population	1967	98.9	0.05	89.3 (85.1–93.2)	67.7 (64.5–69.8)	105 (101–108)	151 (145–155)	219 (215–224)	267	
Males	964	99.4	0.05	86.9 (81.2–92.7)	68.4 (64.6–72.0)	102 (96.9–107)	151 (143–159)	221 (215–228)	264	0.675^a^
Females	1003	99.5	0.05	91.6 (86.0–97.0)	66.9 (63.1–70.8)	107 (102–111)	150 (143–156)	219 (211–223)	267	
7–17 years	622	96.6	0.05	65.4 (57.1–73.9)	61.3 (56.8–64.9)	96.1 (90.5–100)	137 (129–147)	215 (204–222)	262	< 0.001***^b^
7–11	342	95.9	0.05	59.4 (49.1–70.3)	59.9 (51.7–65.3)	95.3 (89.1–106)	132 (125–146)	207 (194–218)	262	0.323^c^
12–17	280	97.5	0.05	73.7 (62.0–86.5)	63.5 (56.4–68.6)	96.4 (89.3–102)	144 (131–160)	221 (212–237)	259	
≧ 18 years	1345	100	21.5	103 (101–106)	70.4 (67.7–74.2)	109 (105–114)	155 (150–161)	222 (217–228)	267	
18–39	370	100	27.3	105 (99.9–110)	73.7 (67.0–80.9)	110 (103–119)	152 (142–162)	218 (209–227)	267	0.781^c^
40–64	591	100	21.5	103 (98.9–108)	71.5 (65.0–77.8)	107 (101–136)	160 (148–169)	227 (218–234)	264	
≧ 65	384	100	21.9	101 (95.7–106)	68.1 (61.6–72.9)	111 (104–117)	155 (144–163)	220 (206–229)	262	< 0.001***
BuP										
Total population	1967	99.7	0.05	5.31 (5.13–5.47)	3.56 (3.43–3.80)	5.71 (5.48–5.92)	8.82 (8.48–9.13)	14.2 (13.9–14.6)	70.2	
Males	964	99.9	0.05	5.35 (5.13–5.61)	3.58 (3.34–3.89)	5.65 (5.35–6.01)	8.83 (8.39–9.31)	14.5 (14.0–14.9)	70.2	0.939
Females	1003	99.7	0.05	5.26 (5.01–5.51)	3.54 (3.36–3.86)	5.73 (5.47–6.04)	8.80 (8.35–9.25)	13.9 (13.3–14.4)	21.5	
7–17 years	622	99.0	0.05	4.36 (4.06–4.66)	2.98 (2.75–3.14)	4.67 (4.35–5.03)	7.71 (7.23–8.42)	13.9 (12.8–14.5)	70.2	< 0.001***
7–11	342	98.8	0.05	4.22 (3.79–4.64)	2.85 (2.51–3.10)	4.49 (4.07–5.19)	7.62 (7.05–8.57)	14.0 (12.6–15.0)	48.6	0.424
12–17	280	99.3	0.05	4.55 (4.13–5.02)	3.11 (2.75–3.35)	4.76 (4.41–5.22)	7.97 (6.94–8.83)	13.6 (12.3–14.5)	70.2	
≧ 18 years	1345	100	0.65	5.81 (5.60–6.01)	4.09 (3.89–4.20)	6.16 (5.88–6.43)	9.21 (8.85–9.56)	14.3 (13.9–14.7)	17.5	
18–39	370	100	1.47	6.49 (6.17–6.84)	4.55 (4.27–5.07)	6.75 (6.32–7.09)	9.39 (8.81–10.3)	14.6 (13.6–15.7)	17.5	0.001**
40–64	591	100	0.65	5.67 (5.39–5.97)	3.89 (3.43–4.13)	5.92 (5.54–6.36)	9.10 (8.60–9.73)	14.3 (13.8–14.8)	16.7	
≧ 65	384	100	0.67	5.41 (5.06–5.77)	3.76 (3.51–4.12)	5.85 (5.26–6.26)	8.87 (8.02–9.69)	14.2 (13.2–14.9)	17.5	< 0.001***^d^

^a^ Comparison of the sex groups by Mann–Whitney *U* test. *p < 0.05, **p < 0.01, ***p < 0.001.

^b^ Comparison of the 7–17 years group and ≧ 18 years groups by Mann–Whitney *U* test.

^c^ Comparison of the age groups by Mann–Whitney *U* test (7–17 years group)/Kruskal–Wallis test (≥ 18 years group).

^d^ Comparison of five age groups by Kruskal–Wallis test.

The highest geometric mean concentration was observed for MeP (357 μg/L), followed by PrP (89.3 μg/L), EtP (34.2 μg/L), and BuP (5.31 μg/L). The distributions of paraben concentrations were further stratified by gender, age, and region.

The geometric mean concentration of BuP was higher among male participants than among female participants (male vs. female = 5.35 vs. 5.26 μg/L). In addition, the geometric mean concentrations of MeP, EtP, and PrP were higher in female than male (male vs. female: MeP, 356 vs. 359 μg/L; EtP, 33.6 vs. 34.7 μg/L; PrP, 86.9 vs. 91.6 μg/L). However, Mann–Whitney *U* test revealed that this gender disparity was not statistically significant.

### 3.3 Distribution of Urinary Paraben Concentration by Age Group

Participants were divided into five age groups: 7–11 (*n* = 342), 12–17 (*n* = 280), 18–39 (*n* = 370), 40–64 (*n* = 591), and ≥ 65 years age groups (*n* = 384) (Table 2 and Figure 1). Among adults, the median concentrations of MeP, EtP, PrP, and BuP were 414 (IQR = 271–612), 42.0 (IQR = 25.5–63.4), 109 (IQR = 70.4–155), and 6.16 μg/L (IQR = 4.09–9.21), respectively. Among minors, the corresponding median concentrations of MeP, EtP, PrP, and BuP were 378 (IQR = 234–565), 38.0 (IQR = 21.4–60.2), 96.1 (IQR = 61.3–137), and 4.67 μg/L (IQR = 2.98–7.71), respectively. Adults had significantly higher paraben concentrations than did minors (*p* < 0.001).

**FIGURE 1:**
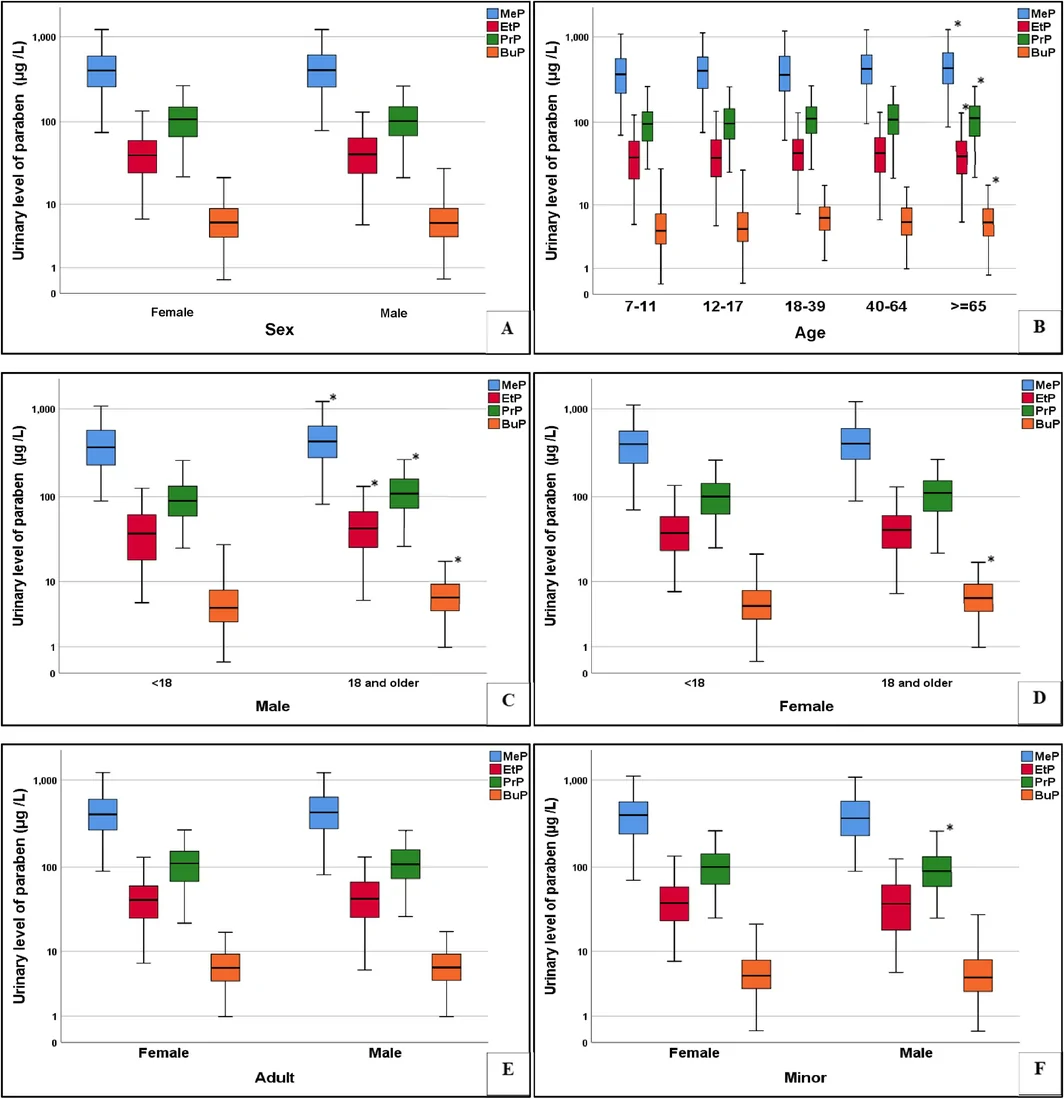
Urinary level of parabens in Taiwanese with different sex and age (*p < 0.05).

The geometric mean concentration of MeP was higher among participants aged ≥ 65 years (425 μg/L) than among those aged 18–39 (407 μg/L) or 40–64 years (360 μg/L; *p* = 0.004). Conversely, the geometric mean concentration of BuP was significantly lower among participants aged ≥ 65 years (37.5 μg/L) than among those aged 18–39 (40.4 μg/L) or 40–64 years (40.2 μg/L; *p* = 0.001).

Overall, significant differences in urinary paraben concentrations were observed across the five age groups.

### 3.4 Distribution of Urinary Paraben Levels by Residential Area

Participants were divided into the following five region‐based groups: northern (*n* = 674), central (*n* = 429), southern (*n* = 522), eastern (*n* = 226), and remote islands (*n* = 116) (Table S1). Urinary concentrations of the parabens were highest in the remote islands and lowest in the eastern and central Taiwan.

The geometric mean concentration of MeP was highest in the remote islands (410 μg/L), followed by southern (375 μg/L), northern (354 μg/L), eastern (344 μg/L), and central (337 μg/L) Taiwan. Similarly, the highest concentration of EtP was observed in the remote islands (42.2 μg/L), followed by northern (35.1 μg/L), southern (34.5 μg/L), central (33.6 μg/L), and eastern (28.6 μg/L) Taiwan. The highest concentration of PrP was detected in the remote islands (104 μg/L), followed by southern (92.2 μg/L), northern (88.3 μg/L), eastern (87.3 μg/L), and central (84.8 μg/L) Taiwan. Last, the highest concentration of BuP was found in the remote islands (5.59 μg/L), followed by northern (5.49 μg/L), central (5.26 μg/L), southern (5.20 μg/L), and eastern (4.99 μg/L) Taiwan. However, no statistically significant regional differences were observed in urinary concentrations of the parabens across these five regions.

### 3.5 Comparison of RV95 Urinary Paraben Concentrations in Different Countries

The concentrations of the parabens in this study were compared with data from biomonitoring programs in other countries (Figure 2). Overall, concentrations of the four parabens in this study were higher than those reported in most other countries. The median concentration of MeP in Taiwan was substantially higher than those in the United States, Canada, South Korea, China, and several European countries. The median concentration of MeP in this study was 404 μg/L, which was significantly higher than that in the United States (28.2 μg/L), Canada (7.8 μg/L), South Korea (34.6 μg/L), China (35.5 μg/L), Germany (39.8 μg/L), Poland (39.8 μg/L), and Belgium (16.1 μg/L). Similarly, the median concentrations of EtP and PrP were higher in Taiwan than in the United States, Canada, and other European countries. BuP concentration and detection rates were also higher in Taiwan compared to other countries, as evidenced by the biomonitoring results in other countries [20, 21, 22, 23, 24, 25, 26, 27, 28] (Figure 2, Table 3).

**FIGURE 2:**
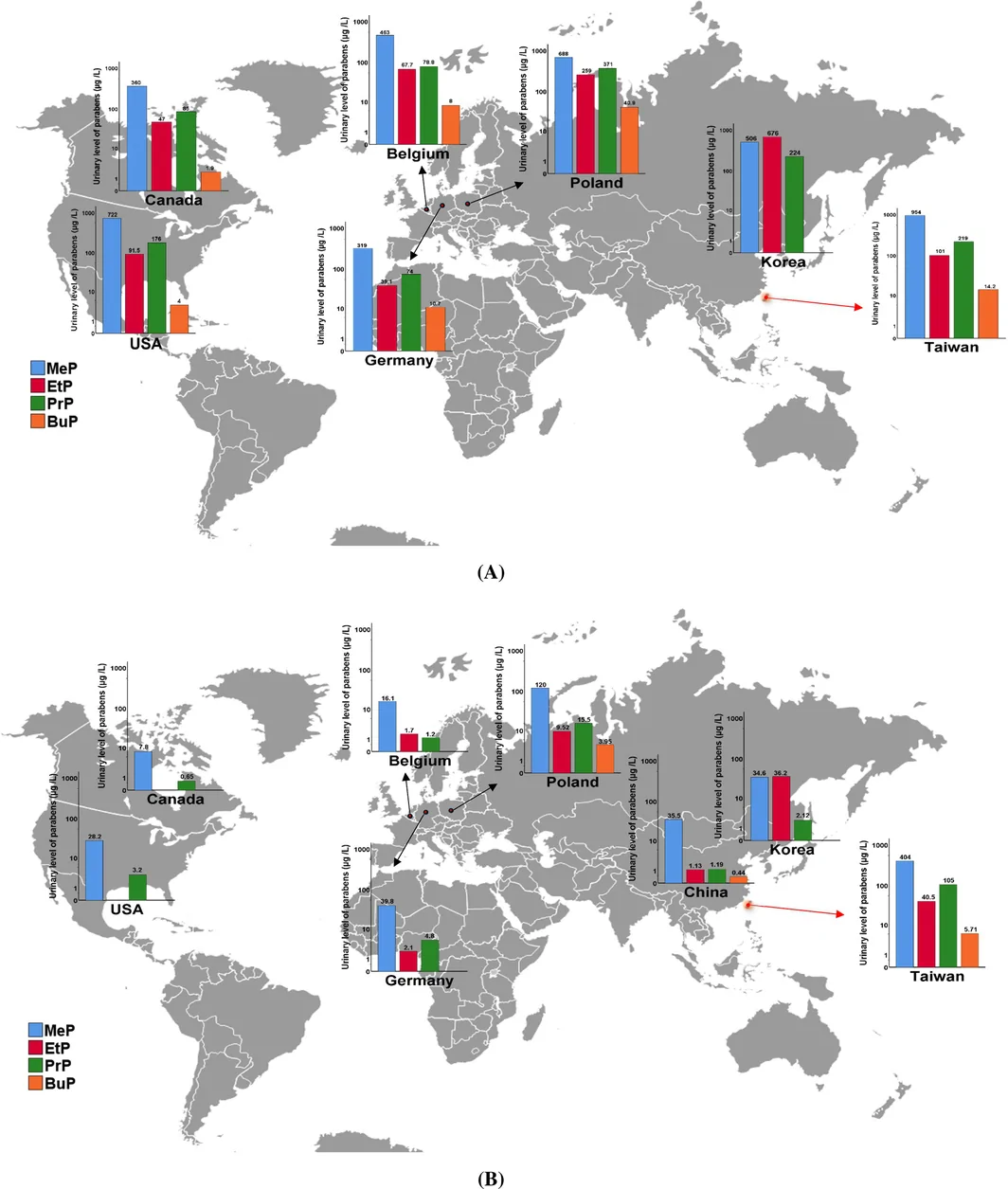
International comparison of (A) 95th percentile levels and (B) median levels among different studies for urinary paraben concentrations (μg/L). Taiwan, this study (*n* = 1967), United States (NHANES, 2015–2016, *n* = 2651), Canada (CHMS, 2018–2019, *n* = 2531), Korea (KoNEHS, 2015–2017, *n* = 3779), Germany (GESB, 1995–2012, *n* = 660), Poland (2014–2019, *n* = 511), Belgium (2013, *n* = 261), and China (2016–2018, *n* = 1405).

**TABLE 3:** Comparison of RV95 (95% CI) values among different national surveys for urinary paraben levels from different countries (μg/L).

Country	RV95; TESTs, Taiwan^a^		RV95; CHMS cycle 6, Canada^b^						RV95; NHANES, USA^c^			RV95; GerES V, Germany^d^			
Year	2013–2016		2018–2019						2015–2016			2014–2017			
Age (years)	7–17	≥ 18	3–5	6–11	12–19	20–39	40–59	60–79	6–11	12–19	≥ 60	3–5	6–10	11–13	14–17
*n*	622	1345	512	498	504	332	343	342	415	405	1690	99	166	103	149
MeP	892 (829–962)	971 (938–991)	120 (< LOD–310)	190 (90–280)	450 (66–830)	580 (130–1000)	240 (180–290)	570 (230–920)	512 (256–909)	670 (287–1300)	727 (647–807)	994 (NA)	659 (NA)	115 (NA)	516 (NA)
EtP	101 (93.5–104)	103 (98.7–107)	3.2 (2.2–4.1)	4.9 (2.2–7.5)	36 (< LOD–85)	47 (2.3–92)	58 (16–100)	50 (22–78)	15.2 (5.10–38.1)	28.2 (6.30–62.9)	117 (75.7–146)	3.92 (NA)	7.64 (NA)	11.6 (NA)	17.4 (NA)
PrP	207 (194–218)	222 (217–228)	17 (0.94–32)	64 (11–120)	76 (27–130)	180 (< LOD–440)	69 (29–110)	140 (57–230)	69.7 (43.9–86.8)	221 (48.8–424)	182 (157–227)	123 (NA)	31.6 (NA)	5.71 (NA)	22.0 (NA)
BuP	13.9 (12.8–14.5)	14.3 (13.9–14.7)	17 (< LOD–49)	0.32 (< LOD–0.39)	0.73 (< LOD–1.5)	0.93 (< LOD–4.7)	1.7 (< LOD–4.1)	5.2 (< LOD–11)	0.30 (0.20–0.80)	1.50 (0.30–4.00)	5.00 (2.00–12.2)				

*Note:* RV95 = P95 as reference value.

^a^ This study.

^b^ Health Canada (2021): Sixth report on human biomonitoring of environmental chemicals in Canada. Minister of Health, Ottawa, ON. Available: https://www.canada.ca/en/health‐canada/services/environmental‐workplace‐health/reports‐publications/environmental‐contaminants/sixth‐report‐human‐biomonitoring/page‐3.html#s9‐2.

^c^ National Health and Nutrition Examination Survey NHANES, 2015–2016.

^d^ German Environmental Survey, GerES 2014–2017. Murawski et al. 2021. https://doi.org/10.1016/j.envres.2020.110502.

An age‐stratified analysis revealed that the median concentration of MeP among children aged 7–11 years in Taiwan was > 10 times higher than that among children aged 6–11 years in the United States (17.2 μg/L), Canada (4.7 μg/L), and South Korea (26.6 μg/L) and that among children aged 6–10 years in Germany (7.26 μg/L) (Table S2). The geometric mean concentration of MeP among adolescents in Taiwan was 8–58 times higher than that among adolescents in other countries. MeP concentrations were 304 μg/L among adolescents aged 12–17 years in Taiwan, 40.5 μg/L among adolescents aged 12–19 years in the United States, 8.0 μg/L among adolescents aged 12–19 years in Canada, 16.3 μg/L among adolescents aged 12–18 years in South Korea, and 8.43 μg/L among adolescents aged 14–17 years in Germany.

The geometric mean concentration of EtP among children and adolescents in South Korea (children: 10.5 μg/L; adolescents: 11.9 μg/L) was approximately half that among children and adolescents in Taiwan (children: 23.9 μg/L; adolescents: 26.3 μg/L). In addition, children and adolescents in Taiwan exhibited much higher EtP concentrations than did those in Germany (children: 0.92 μg/L; adolescents: 0.70 μg/L).

The 95th percentile PrP concentrations among children in Taiwan (207 μg/L) were more than twice those among children in the United States (69.7 μg/L) and South Korea (99.4 μg/L).

The 95th percentile PrP concentration among adolescents in Taiwan (221 μg/L) was identical to that among adolescents aged 12–19 years in the United States (221 μg/L). However, the geometric mean concentration of PrP was > 20 times higher in Taiwan (73.7 μg/L) than in the United States (3.09 μg/L). The mean concentrations of PrP among children and adolescents in Taiwan (children: 59.4 μg/L; adolescents: 73.7 μg/L) were higher than those among children and adolescents in Canada (children: No Publication; adolescents: 1.0 μg/L), South Korea (children: 1.7 μg/L; adolescents: 2.0 μg/L), and Germany (children: 0.55 μg/L; adolescents: 0.75 μg/L). The 95th percentile BuP concentrations among children and adolescents in Taiwan (children: 14.0 μg/L; adolescents: 13.6 μg/L) were 46 times and 9 times higher, respectively, than those among children and adolescents in the United States (children: 0.30 μg/L; adolescents: 1.50 μg/L) (Table S2).

Among adults, the geometric mean concentration of MeP was more than seven times higher in Taiwan (360–425 μg/L) than in the United States (52.2 μg/L). Marked disparity was also found for the 95th percentile concentration. The concentration of MeP in Taiwan was also considerably higher than that in Canada (240–580 μg/L). The median concentration of EtP in Taiwan (39.4–42.9 μg/L) was comparable to that in South Korea (36.2 μg/L) and was considerably higher than those in the United States, Canada, and Germany. The median concentration of PrP was tens of times higher in Taiwan (107–111 μg/L) than in the United States (4.00 μg/L), Canada (0.54–1.3 μg/L), or South Korea (2.1 μg/L) (Table S2).

The 95th percentile PrP concentrations were 207–222 μg/L in Taiwan, 182 μg/L in the United States, and 69–180 μg/L in Canada [20, 21, 27, 28, 29, 30] (Table 3).

An examination of RV95s across different age groups in Taiwan revealed a significant correlation only between the MeP concentration and age, which were positively correlated. RV95s for MeP in Taiwan were 1–2 times higher than those in the United States, Canada, and other European countries, particularly among children and adolescents. RV95s for EtP among children and adolescents in Taiwan were several tens of times higher than those in other Western countries. RV95s for EtP were 1–4 times lower among children and adolescents in Taiwan than among children and adolescents in South Korea. This discrepancy may be attributed to the dietary habits of children and adolescents in South Korea. A US study also reported significant gender‐ and race‐based variations in paraben concentrations across diverse populations [31].

### 3.6 Association of Paraben Exposure With Demographic Variables

The association between demographic variables and the urinary paraben concentrations was examined using multiple regression analysis. Age was positively correlated with the urinary concentrations of MeP, EtP, and PrP. The use of personal care products was significantly and positively associated with the urinary concentrations of MeP (*β* = 23.8, 95% CI = 3.43–44.1). This finding suggests that paraben exposure in Taiwan is likely driven could be primarily by personal care product use. However, and other potential factors sources, such as dietary sources, should also be considered (Table 4).

**TABLE 4:** Multiple regression between paraben exposure and variables (all participants).

Variable	MeP				EtP				PrP				BuP			
	*B* (95% CI)	*β*	*p*	VIF	*B* (95% CI)	*β*	*p*	VIF	*B* (95% CI)	*β*	*p*	VIF	*B* (95% CI)	*β*	*p*	VIF
Constant	76.1 (47.2, 105)		< 0.001		9.47 (6.14, 12.8)		< 0.001		27.2 (21.3, 33.1)		< 0.001		1.78 (1.23, 2.33)		< 0.001	
Sex (male)	4.83 (−13.7, 23.4)	0.009	0.610	1.120	1.53 (−0.61, 3.67)	0.027	0.160	1.119	−**4.12** **(**−**7.97**, −**0.27)**	−**0.036**	**0.036**	1.117	0.19 (−0.168, 0.55)	0.023	0.280	1.119
Age (years)	**0.68** **(0.27, 1.08)**	**0.064**	**0.001**	1.209	−0.01 (−0.06, 0.04)	−0.008	0.705	1.216	0.03 (−0.06, 0.11)	0.012	0.508	1.216	0.0005 (−0.01, 0.01)	0.003	0.900	1.216
BMI (overweight)^a^	−7.33 (−26. 9, 12.2)	−0.014	0.462	1.149	1.03 (−1.22, 3.28)	0.018	0.371	1.148	−1.14 (−5.21, 2.92)	−0.010	0.581	1.149	−0.08 (−0.45, 0.29)	−0.009	0.679	1.149
Education (junior)	−6.00 (−25.0, 13.0)	−0.011	0.536	1.016	−0.49 (−2.67, 1.70)	−0.008	0.663	1.016	1.92 (−2.02, 5.87)	0.016	0.340	1.016	0.19 (−0.17, 0.55)	0.021	0.298	1.016
Smoking (yes)	2.43 (−26.9, 31.8)	0.003	0.871	1.191	−0.39 (−3.77, 2.99)	−0.005	0.820	1.191	4.42 (−1.68, 10.5)	0.025	0.155	1.190	−0.12 (−0.67, 0.44)	−0.009	0.679	1.191
Drinking (yes)	−4.59 (−51.7, 42.5)	−0.003	0.848	1.051	1.15 (−4.28, 6.57)	0.008	0.678	1.050	3.65 (−6.14, 13.4)	0.012	0.465	1.050	0.45 (−0.44, 1.34)	0.020	0.322	1.050
PCPs usage (high)^b^	**23.8** **(3.43, 44.1)**	**0.041**	**0.022**	1.017	−0.88 (−3.23, 1.47)	−0.014	0.462	1.019	−1.14 (−5.37, 3.09)	−0.009	0.597	1.019	−0.17 (−0.56, 0.21)	−0.017	0.383	1.019
MeP (μg/L)					**0.02** **(0.02, 0.03)**	**0.210**	< **0.001**	1.648	**0.09** **(0.08, 0.09)**	**0.381**	< **0.001**	1.427	**0.002** **(0.002, 0.003)**	**0.140**	< **0.001**	1.691
EtP (μg/L)	**1.74** **(1.36, 2.13)**	**0.192**	< **0.001**	1.504					**0.61** **(0.53, 0.68)**	**0.297**	< **0.001**	1.390	**0.021** **(0.013, 0.028)**	**0.135**	< **0.001**	1.542
PrP (μg/L)	**1.98** **(1.78, 2.18)**	**0.445**	< **0.001**	1.667	**0.19** **(0.16, 0.21)**	**0.380**	< **0.001**	1.781					**0.023** **(0.019, 0.027)**	**0.311**	< **0.001**	1.877
BuP (μg/L)	**6.56** **(4.20, 8.92)**	**0.110**	< **0.001**	1.325	**0.76** **(0.49, 1.03)**	**0.116**	< **0.001**	1.325	**2.79** **(2.32, 3.27)**	**0.209**	< **0.001**	1.258				

*Note:* All *p*‐values below 0.05 are shown in bold type. These analyses included unstandardized coefficient (*B*), 95% confidence interval (CI), standardized coefficient (*β*), and *p*‐value.

^a^ BMI ≧ 24.

^b^ Use more than one kind of personal care products.

### 3.7 Risk Assessment

Overall, the median DI values of MeP, EtP, PrP, and BuP were 38.7, 4.89, 20.8, and 1.71 μg/kg/day, respectively. Among minors, the median DI values of MeP, EtP, PrP, and BuP were 40.0, 4.94, 20.6, and 1.65 μg/kg/day, respectively. Among adults, the corresponding median DI values of MeP, EtP, PrP, and BuP were 39.4, 4.86, 20.9, and 1.73 μg/kg/day. Overall, DI values were comparable between adults and minors. Overall, median DI values were well within the average DI health‐based guidance values (MeP+ EtP: 10 mg/kg/day) (Table S3).

The maximum HQ for the combined DI MeP and EtP among all participants was 0.137, which was substantially below the threshold of 1 (Table S3, Figure S1). This indicates that exposure levels of MeP and EtP were well below the RfDs for adverse health effects (10 mg/kg/day). At the 95th percentile, daily MeP and EtP intake accounted for only 1.7% of the HI, and in the 50th percentile, daily MeP and EtP intake accounted for 0.4% of the HI.

In contrast, PrP had the highest HQ among the four parabens at 28.6, where BuP had the lowest maximum at 2.19. Among all 1967 participants, 55.4% of the HI value exceeded the threshold value of 1 (Figure 3). The median HI (calculated as the sum of the corresponding HQs for each paraben) was 1.14, with a 95th percentile HI of 4.07 and a maximum HI of 30.9.

**FIGURE 3:**
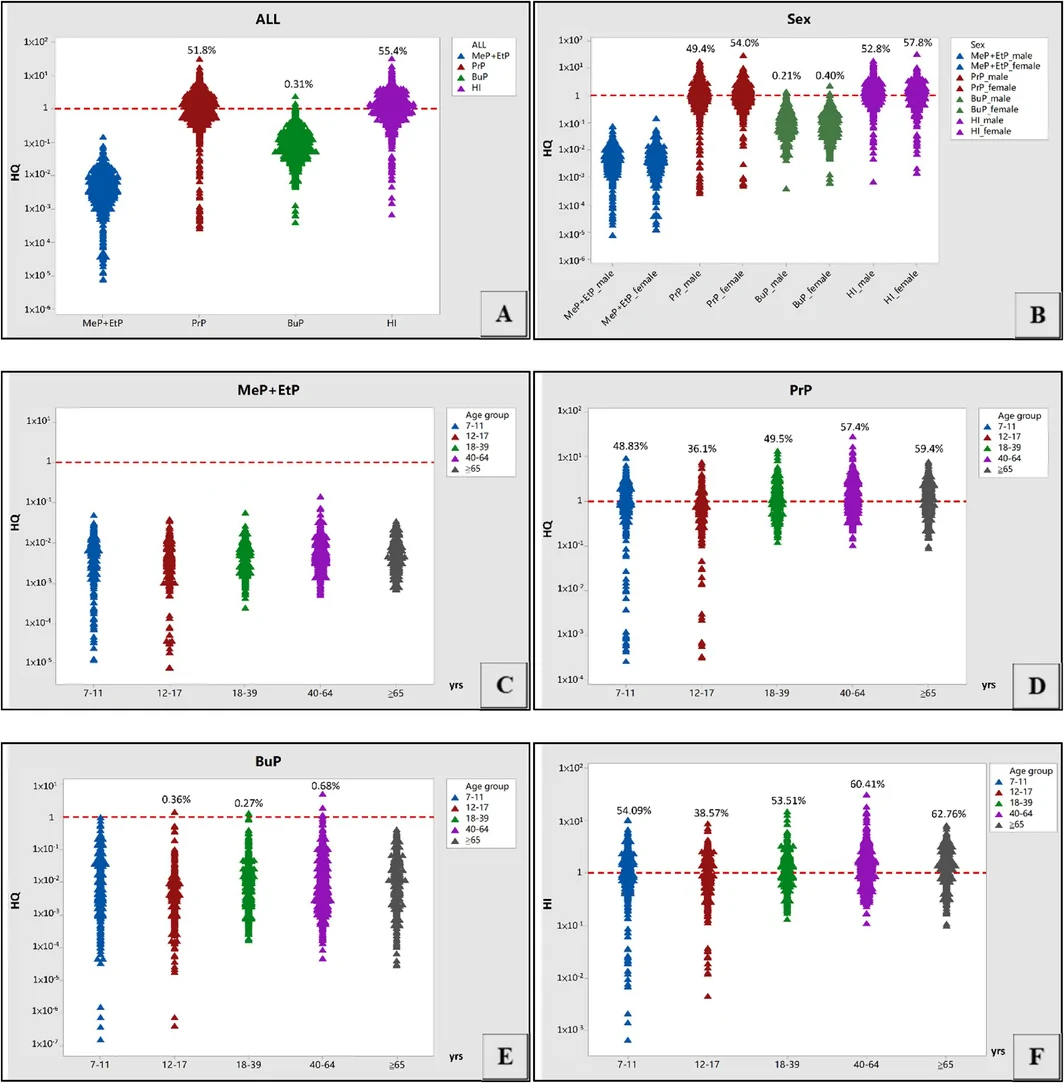
Hazard quotient in Taiwanese due to paraben exposure ((A) all participants; (B) sex; (C–F) age group).

## 4 Discussion

The findings of this study suggest that parabens are widely detected in the urine of the general population in Taiwan, suggesting ubiquitous exposure. Detection frequencies among minors (67.4%–100%) were significantly lower than those among adults (100%). It is recognized that *p*‐hydroxybenzoic acid (PHBA) and its conjugates are metabolites of parabens; however, extensive evidence indicates that PHBA is a nonspecific and quantitatively unreliable biomarker for congener‐specific paraben exposure. First, PHBA is not exclusive to parabens—it can originate from multiple sources, including dietary intake and endogenous metabolism [32]. Studies show that only a limited fraction of parabens is converted to PHBA, with substantial inter‐individual and inter‐compound variability, making back‐calculation of exposure highly uncertain [32]. Second, PHBA cannot differentiate between different individual paraben compounds, which vary considerably in estrogenic activity and toxicological potency. Aggregating all parabens into a single PHBA measure would obscure these differences and potentially misrepresent health risks [33]. Third, epidemiological and toxicokinetic evidence suggests that a substantial portion of urinary PHBA arises from non‐paraben sources, further limiting its specificity as an exposure metric.

In this study, the order of paraben concentrations was MeP > PrP > EtP > BuP. Furthermore, the urinary concentrations of all four parabens were positively correlated with participant age. Despite the absence of statistically significant differences in paraben concentrations between different genders or regions, notable differences were observed across five age groups. Among adults, the urinary concentrations of MeP increased with age, but BuP concentrations decreased with age.

It is evident that the utilization of personal care products is recognized as a major contributor to paraben exposure. However, to date, no comprehensive study has been conducted to investigate the role of these products as a potential origin of parabens. A notable correlation was observed between personal care product use and the urinary concentrations of the parabens in the present study. This finding suggests that even products traditionally considered safe based on dermal exposure assessments may pose potential risks if used frequently. This has the potential to further compound the risk of adverse health consequences.

Adults are typically exposed to a greater number of products and medications than are minors, resulting in greater exposure for adults. A number of studies have indicated that discrepancies in urinary paraben concentrations across countries may be attributable to variations in the paraben composition of personal care products or distinct washing and dietary practices. The median urinary EtP concentrations in this study were similar to those reported in Korea, where the consumption of spicy fermented food (i.e., kimchi) has been identified as a significant factor influencing exposure [24]. We recognize that urine samples in the present study were collected during 2013–2016, and that paraben use has declined in several developed countries, including the US and EU, following regulatory restrictions implemented during the 2000s and 2010s. Besides, urinary parabens were quantified using isotope‐dilution LC–MS/MS with method validation and quality control procedures performed in accordance with guidelines from the European Medicines Agency for bioanalytical methods. The use of stable isotope‐labeled internal standards helps compensate for matrix effects, including ion suppression and ion enhancement, thereby improving quantification accuracy. Although the urinary concentrations of MeP and PrP observed in the present study were higher than those reported in several international biomonitoring programs, differences in hydrolysis protocols, analytical workflows, calibration ranges, and laboratory‐specific procedures may contribute to variability across studies, thus the relatively higher concentrations observed here should be interpreted more carefully. Previous studies have also shown that the efficiency of enzymatic hydrolysis of paraben conjugates can vary depending on factors such as enzyme source, enzyme activity during preparation, and the amount of enzyme used during sample preparation. In addition, the presence of high proportion of methanol in urine samples prior to incubation may help prevent unintended hydrolysis of the ester bond of methyl paraben [34]. Because no universally standardized analytical method currently exists for determining urinary parabens, inter‐study comparisons should be interpreted cautiously.

Therefore, comparisons with more recent biomonitoring data from other countries should be interpreted with caution. In the event of an inter‐country comparison of HBM data, the interpretation of results may be influenced by the consideration of the following factors: The presence of methodological discrepancies (for instance, the allocation of values to measurements that fall below the limit of detection, the employment of field blanks, and the utilization of analytical approaches) has been identified. The higher urinary paraben levels observed in this study likely reflect temporal differences in sampling periods and regulatory timelines rather than inherently higher current exposure in Taiwan.

By calculating the cumulative risk, we found considerable risk in participants. Maximum HQs ranged from 0.137 to 28.6, and the HI was 30.9. Both the values exceeded the cumulative thresholds. In addition, we concluded that more than half of the participants exhibited HI values exceeding 1. This indicates potential concern under protective assumptions, particularly given the conservative and uncertain toxicological inputs (e.g., the NOEL for PrP). However, this should not be interpreted as evidence of a definitive or significant health risk. In the absence of official health‐based guidance values for PrP and BuP, we applied a conservative screening‐level approach using a NOEL‐based point of departure combined with an uncertainty factor of 100 to derive values for the HQ/HI framework, consistent with previous assessments [7, 15, 18]. PrP was identified as the most significant influencing factor of individual HI in the study population. Despite a lower DI of PrP than that of MeP, the calculated risk was the highest. This result indicates that PrP exposure represents the most significant risk of paraben exposure for the public in Taiwan.

The term RV95 (reference value at the 95th percentile) is employed in the field of statistics to denote statistical benchmarks for substances present within the human body. These benchmarks are typically associated with pollutants present within urine and represent the concentrations that are observed in a healthy population. However, it should be noted that these values are not adjusted for creatinine. This is because creatinine itself exhibits significant variability, which is influenced by factors such as muscle mass, age, and hydration levels. To accurately assess exposure, urinary biomarker data is typically adjusted for creatinine to account for dilution of the spot sample. The 95th percentiles of the MeP DI values in this study (151 μg/kg/day) were higher than those among adults in China (1.49–1.56 μg/kg/day) [35] and the United States (0.358 μg/kg/day) [36]. The 95th percentiles of the DI values of EtP in the present study (20.4 μg/kg/day) were higher than those in China (0.166–0.174 μg/kg/day) and the United States. The 95th percentiles of the DI values of PrP were higher in the present study in Taiwan (74.9 μg/kg/day) than those in China (0.426–0.447 μg/kg/day) and the United States (0.098 μg/kg/day). Furthermore, the 95th percentiles of the DI values of BuP were higher in the present study in Taiwan (7.42 μg/kg/day) than those in China (0.087–0.090 μg/kg/day) and the United States (0.0013 μg/kg/day). Additionally, with regard to the acceptable DI, the DI values of MeP and EtP for our entire population were considerably lower than the acceptable daily standard value of 10 mg/kg/day recommended by the World Health Organization. Furthermore, the DI of BuP was below the RfD of 0.02 mg/kg/day. However, in > 50% of the population, the DI of PrP exceeded the RfD. Only four parabens were examined in this study; consequently, the calculated exposures may be underestimated. The present study findings support the findings of Moos et al. [15], who identified PrP as the primary source of paraben exposure. The purpose of the tools mentioned above is to make it easier to compare the body burden in different places with Taiwan. They are very useful for showing if a certain level of exposure is higher than the background level, for example, in the case of accidents. RVs are constantly checked and updated when new information is available.

The findings of this study indicate that the estimated DI values of parabens are higher among adults than among children. This discrepancy may be attributed to the differing utilization of personal care products and dietary habits between adults and children. The RV95 is a tool that helps with public health and environmental management by identifying potential risks linked to parabens exposure. It helps to identify places where humans and the environment might be exposed to potentially harmful parabens. Consequently, this implies that measures may be taken to mitigate these risks. Additionally, the Taiwan Food and Drug Administration has played a role in reducing the source of exposure in children by stipulating the relevant total amount of additives.

In another study, which analyzed data from the TEST 2013 cohort, participants with elevated cumulative levels of parabens exhibited an increased risk of elevated PrP concentrations (> 75th percentile concentration) [7]. We also note that Taiwan's biomonitoring data were collected during 2013–2016, a period preceding the implementation of stricter paraben regulations by the Taiwan Food and Drug Administration (TFDA) in 2017–2018. In contrast, several European countries had already revised paraben‐related cosmetic regulations earlier (e.g., in 2014). These regulatory timing differences may partially explain the relatively higher paraben levels observed in Taiwan during the study period. This may contribute to a greater number of sources of exposure.

The principal strength of this study is its large sample size, which enabled a robust analysis. The data employed in this study were derived from a representative sample of individuals who were aged between 7 and 97 years and were from the general population in Taiwan. The exposure profile of the study participants reflects that of the general population in Taiwan. The study yielded valuable insights into the urinary concentrations of the parabens of the Taiwanese population, uncovering significant differences based on sex, age, and region. Additionally, comparisons were made with several other countries. The RVs obtained for the four parabens in the present study may be used in future public health studies. To our knowledge, this is the first study to establish population‐based RVs for multiple parabens in Taiwan using a nationwide survey. The Taiwanese population, which is predominantly of Han Chinese ancestry and characterized by distinct dietary patterns and consumer behaviors, provides important context for exposure assessment that may not be captured in Western cohorts. In addition, this study integrates RV derivation with cumulative risk assessment, including estimation of DI, HQ, and HI, and places the findings in an international context by comparison with contemporaneous biomonitoring studies. A comparison of the results with population RVs is warranted.

This study has several limitations. It is recognized that the recruitment process took place during community‐based and school health examination events, which may have resulted in the preferential attraction of individuals with higher health awareness or socioeconomic status, thereby introducing a potential element of self‐selection bias. This limitation is inherent to large‐scale biomonitoring surveys relying on voluntary participation and is now explicitly acknowledged in the revised manuscript. However, the use of a stratified, multistage sampling framework consistent with NAHSIT, combined with wide geographic coverage and inclusion of all age groups, was intended to approximate representativeness of the general Taiwanese population. Population weighting was not applied in the present analysis, as the primary objective was to establish biomonitoring RVs rather than population prevalence estimates. As a cross‐sectional study, the one‐time measurements were conducted retrospectively, thereby providing an accurate reflection of the participants' presurvey exposure. The concentrations of parabens in urine primarily reflect short‐term exposure because parabens are rapidly metabolized and excreted, with elimination half‐lives generally being less than 24 h [37]. Therefore, single spot urine samples may not fully capture long‐term exposure variability. However, urinary biomarkers are widely used in biomonitoring studies [38] because parabens are continuously and repeatedly encountered through daily use of personal care products, cosmetics, and other common sources [38]. As a result, spot urine measurements are considered appropriate for characterizing population‐level exposure patterns rather than individual long‐term exposure [39]. It is acknowledged that for the general population, the time between exposure and sample collection may not be easily quantifiable. It is a general rule that population‐level biomonitoring studies include large sample sizes in order to compensate for such limitations [40]. In addition, the detailed quantitative information on specific PCP products, brands, paraben content, usage frequency, or amount was not available in the TEST dataset; therefore, direct assessment of product‐specific paraben exposure was not feasible. The PCP use variable was included as a categorical covariate to adjust for general exposure patterns rather than to estimate dose–response relationships. The observed association with urinary paraben levels is consistent with prior literature and should be interpreted as supportive but not quantitative. Furthermore, comparisons were made between paraben concentrations in different countries. However, this methodology was not optimal due to discrepancies in dietary patterns, environmental exposures, and varying time frames across countries.

## 5 Conclusion

This study collected and analyzed the urinary concentrations of selected parabens in a representative cohort of 1967 individuals across Taiwan. The results are based on human biomonitoring data from the TEST, providing valuable information on the urinary concentrations of the parabens in the general Taiwanese population. Background urinary paraben concentrations, which are an invaluable tool for identifying the potential sources of exposure, were established in this study for the general Taiwanese population. Compared with RV95 concentrations in other countries, those in Taiwan are relatively high. Paraben exposure levels and potential health effects should be acknowledged. Over half of the participants had HI values above 1, indicating a high risk of exposure to parabens. PrP was the main factor affecting HI in the study population.

## Funding

We would also like to extend thanks to the National Health Research Institutes for their financial support (Grant No.: EM‐114‐PP‐11, EM‐115‐PP‐10, EM‐112‐SP‐01, EM‐112‐SP‐02, NHRI‐EX111‐11116PI), and the Ministry of Science and Technology Council (Grant No.: MOST 110‐2314‐B‐A49A‐543, MOST 110‐2314‐B‐400‐039), and the National Science and Technology Council (Grant No.: NSTC 111‐2314‐B‐A49‐005, NSTC 112‐2314‐B‐A49‐005, NSTC 112‐2314‐B‐400‐006, and NSTC 111‐2314‐B‐400‐013). This work was supported partially by the Research Center for Environmental Medicine, Kaohsiung Medical University, Kaohsiung, Taiwan from The Featured Areas Research Center Program within the framework of the Higher Education Sprout Project by the Ministry of Education (MOE) in Taiwan and by Kaohsiung Medical University Research Center Grant (KMU‐TC114A01‐1).

## Conflicts of Interest

The authors declare no conflicts of interest.

## Supporting information


**Table S1:** Urinary levels of parabens in Taiwanese among different residential area.
**Table S2:** Comparison of urinary level of parabens (μg/L) in general population among different countries.
**Table S3:** Paraben daily intake DI (μg/kg bw/day), hazard quotient (HQ) and hazard index (HI) of all population, minors and adults.
**Figure S1:** The hazard quotient of general Taiwanese by different age group.
**Figure S2:** Distribution of urinary paraben levels in Taiwanese among different sex.

## Data Availability

The data that support the findings of this study are available from the corresponding author upon reasonable request.
