# Supplementary material for: Paraben Exposure in the General Taiwanese Population: Reference Values, Personal Care Products, and Cumulative Risk Assessment
Source: Kaohsiung J Med Sci. 2026 Apr 11:e70208. Online ahead of print. doi: 10.1002/kjm2.70208 (PMC13399792; doi:10.1002/kjm2.70208)

**Table S1. Urinary levels of parabens in Taiwanese among different residential area.**

| Parabens | n | %>LOD | min | GM (95%CI) | Selected percentiles | | | |  | Max |  | p-value^a^ |
| --- | --- | --- | --- | --- | --- | --- | --- | --- | --- | --- | --- | --- |
| (μg/L) |  |  |  |  | 25^th^ (95%CI) | 50^th^ (95%CI) | 75^th^ (95%CI) | 95^th^ (95%CI) |  |  |  |  |
| **MeP** |  |  |  |  |  |  |  |  |  |  |  |  |
| Total population | 1967 | 100 | 0.42 | 357 (344-372) | 257 (248-266) | 404 (385-418) | 603 (581-626) | 954 (921-984) |  | 1211 |  |  |
| Northern | 674 | 100 | 0.42 | 354 (331-377) | 250 (232-274) | 401 (377-427) | 605 (561-649) | 957 (912-987) |  | 1184 |  | 0.432 |
| Central | 429 | 100 | 0.55 | 337 (308-367) | 252 (234-265) | 377 (356-421) | 588 (536-621) | 921 (867-1008) |  | 1204 |  |  |
| Southern | 522 | 96.6 | 0.67 | 375 (351-400) | 268 (245-288) | 414 (377-440) | 610 (564-662) | 951 (888-1006) |  | 1210 |  |  |
| Eastern | 226 | 95.9 | 0.47 | 344 (301-391) | 253 (218-269) | 409 (352-446) | 610 (537-681) | 958 (901-1067) |  | 1211 |  |  |
| Remote island | 116 | 97.5 | 103 | 410 (365-453) | 264 (239-320) | 418 (372-484) | 656 (553-772) | 973 (853-1104) |  | 1134 |  |  |
| **EtP** |  |  |  |  |  |  |  |  |  |  |  |  |
| Total population | 1967 | 98.4 | 0.05 | 34.2 (32.4-35.8) | 24.4 (23.6-25.9) | 40.5 (39.0-42.2) | 62.5 (60.3-64.8) | 101 (98.6-105) |  | 134 |  |  |
| Northern | 674 | 100 | 0.05 | 35.1 (32.3-37.9) | 24.2 (22.7-25.9) | 40.7 (37.9-44.1) | 65.2 (61.8-69.3) | 105 (98.1-109) |  | 134 |  | 0.146 |
| Central | 429 | 100 | 0.05 | 33.6 (30.1-37.5) | 25.4 (23.0-28.4) | 42.4 (38.1-45.3) | 61.0 (57.4-68.2) | 99.5 (91.0-107) |  | 130 |  |  |
| Southern | 522 | 99.0 | 0.05 | 34.5 (31.5-37.6) | 25.4 (23.4-27.0) | 40.1 (37.2-42.7) | 59.7 (56.4-64.4) | 102 (96.1-107) |  | 128 |  |  |
| Eastern | 226 | 98.8 | 0.05 | 28.6 (23.8-34.0) | 22.1 (17.7-25.2) | 36.9 (32.8-42.1) | 58.1 (52.7-65.6) | 94.0 (83.6-101) |  | 124 |  |  |
| Remote island | 280 | 99.3 | 6.86 | 42.2 (37.4-48.1) | 24.6 (22.1-29.3) | 44.3 (37.4-51.2) | 69.8 (57.1-85.9) | 104 (98.1-124) |  | 130 |  |  |

^a^ Comparison of five residential areas by Kruskal–Wallis test

**Table S1. Urinary levels of parabens in Taiwanese among different residential area. (Cont.)**

| Parabens | n | %>LOD | min | GM (95%CI) | Selected percentiles | | | |  | Max |  | p-value^a^ |
| --- | --- | --- | --- | --- | --- | --- | --- | --- | --- | --- | --- | --- |
| (μg/L) |  |  |  |  | 25^th^ (95%CI) | 50^th^ (95%CI) | 75^th^ (95%CI) | 95^th^ (95%CI) |  |  |  |  |
| **PrP** |  |  |  |  |  |  |  |  |  |  |  |  |
| Total population | 1967 | 98.9 | 0.05 | 89.3 (85.1-93.2) | 67.7 (64.5-69.8) | 105 (101-108) | 151 (145-155) | 219 (215-224) |  | 267 |  |  |
| Northern | 674 | 100 | 0.05 | 88.3 (80.8-95.4) | 68.2 (63.7-72.9) | 107 (101-112) | 152 (142-157) | 218 (208-225) |  | 260 |  | 0.722 |
| Central | 429 | 100 | 0.05 | 84.8 (75.4-94.4) | 65.7 (60.9-72.7) | 99.8 (93.2-106) | 146 (138-159) | 219 (209-228) |  | 267 |  |  |
| Southern | 522 | 96.6 | 0.05 | 92.2 (85.6-98.5) | 67.1 (63.0-72.4) | 102 (93.9-107) | 153 (144-162) | 219 (207-229) |  | 262 |  |  |
| Eastern | 226 | 95.9 | 0.05 | 87.3 (74.4-101) | 65.6 (57.5-77.5) | 111 (98.3-119) | 146 (137-167) | 230 (212-238) |  | 262 |  |  |
| Remote island | 116 | 97.5 | 27.6 | 104 (95.2-114) | 74.5 (58.6-90.0) | 110 (98.8-120) | 151 (136-171) | 213 (199-224) |  | 247 |  |  |
| **BuP** |  |  |  |  |  |  |  |  |  |  |  |  |
| Total population | 1967 | 99.7 | 0.05 | 5.31 (5.13-5.47) | 3.56 (3.43-3.80) | 5.71 (5.48-5.92) | 8.82 (8.48-9.13) | 14.2 (13.9-14.6) |  | 70.2 |  |  |
| Northern | 674 | 100 | 0.05 | 5.49 (5.22-5.77) | 3.78 (3.48-4.00) | 5.68 (5.33-6.02) | 8.79 (8.17-9.32) | 13.9 (13.3-14.6) |  | 70.2 |  | 0.948 |
| Central | 429 | 100 | 0.05 | 5.26 (4.87-5.64) | 3.54 (3.23-4.06) | 5.77 (5.38-6.19) | 8.76 (7.92-9.70) | 14.3 (13.4-15.0) |  | 27.9 |  |  |
| Southern | 522 | 99.0 | 0.05 | 5.20 (4.87-5.53) | 3.35 (3.13-3.89) | 5.66 (5.22-6.10) | 8.61 (8.26-9.19) | 14.2 (13.2-14.8) |  | 26.9 |  |  |
| Eastern | 226 | 98.8 | 0.05 | 4.99 (4.41-5.58) | 3.28 (2.87-3.92) | 5.69 (5.03-6.60) | 9.52 (8.50-10.0) | 14.6 (13.5-15.3) |  | 16.9 |  |  |
| Remote island | 280 | 99.3 | 0.98 | 5.59 (4.94-6.30) | 3.75 (2.92-4.36) | 5.80 (5.11-6.94) | 8.69 (7.92-9.82) | 14.3 (13.0-15.9) |  | 16.3 |  |  |

^a^ Comparison of five residential areas by Kruskal–Wallis test.

**Table S2. Comparison of national surveys on the distribution of urinary paraben (μg/L) concentrations in general population groups by age in various countries**

| Paraben |  | TESTs, Taiwan ^a^ | | | | | | | | |  | NHANES, USA^b^ | | | | |  | | CHMS, Canada ^c^ | | | | | | | | | | | |  | | KoNEHS, Korea | | | | |  | | GerES V, Germany | | | | |  | |  | | |
| --- | --- | --- | --- | --- | --- | --- | --- | --- | --- | --- | --- | --- | --- | --- | --- | --- | --- | --- | --- | --- | --- | --- | --- | --- | --- | --- | --- | --- | --- | --- | --- | --- | --- | --- | --- | --- | --- | --- | --- | --- | --- | --- | --- | --- | --- | --- | --- | --- | --- |
| Year |  | 2013-2-16 | | | | | | | | |  | 2015-2016 | | | | |  | | 2018-2019 | | | | | | | | | | | |  | | 2015- 2017 | | | | |  | | 2014–2017 ^f^ | | | | |  | | 2012 ^g^ | | |
| age(yrs) |  | 7-11 |  | 12-17 |  | 18-39 |  | 40-64 |  | ≥ 65 |  | 6-11 |  | 12-19 |  | ≥ 20 | |  | | 3-5 |  | 6-11 |  | 12-19 |  | 20-39 |  | 40-59 |  | 60-79 | |  | 6-11^d^ |  | 12-18 ^d^ |  | 19-86^e^ | |  | 6-10 |  | 11-13 |  | 14-17 | |  | 20-30 | |  |
| n |  | 342 |  | 280 |  | 370 |  | 591 |  | 384 |  | 415 |  | 405 |  | 1690 | |  | | 512 |  | 498 |  | 504 |  | 332 |  | 343 |  | 342 | |  | 884 |  | 900 |  | 3779 | |  | 155 |  | 97 |  | 145 | |  | 60 | |  |
| MeP |  | 275 (364, 892) |  | 304 (401, 918) |  | 360 (358, 933) |  | 407 (422, 961) |  | 425 (428, 986) |  | 17.2 (10.7, 512) |  | 40.5 (32.0, 942) |  | 52.2 (46.8, 823) | |  | | 6.3 (5.6, 120) |  | 4.7 (3.5, 190) |  | 8.0  (5.9, 450) |  | 11  (7.2, 580) |  | 11  (8.7, 240) |  | 18  (19, 570) | |  | 26.6 (NP, 754) |  | 16.3 (NP, 309) |  | NP (34.6, NP) | |  | 7.26 (5.06, 659) |  | 5.23 (4.06, 115) |  | 8.43 (6.1, 516) | |  | NP (42.6, 252) | |  |
| EtP |  | 23.9 (38.2, 100) |  | 26.3 (37.8, 101) |  | 40.2 (42.9, 98.5) |  | 40.4 (42.9, 109) |  | 37.5 (39.4, 98.8) |  | NP (ND, 15.2) |  | NP (ND, 28.2) |  | NP (ND, 117) | |  | | NP (ND, 3.2) |  | NP (ND, 4.9) |  | NP (ND, 36) |  | NP (ND, 47) |  | NP (ND, 58) |  | NP (ND, 50) | |  | 10.5 (NP, 440) |  | 11.9 (NP, 177) |  | NP, (36.2, NP) | |  | 0.92 (0.72, 7.64) |  | 0.70 (0.64, 11.6) |  | 1.47 (1.13, 17.4) | |  | NP  (2.1, 67.3) | |  |
| PrP |  | 59.4 (95.3, 207) |  | 73.7 (96.4, 221) |  | 105 (110, 218) |  | 103 (107, 227) |  | 101 (111, 220) |  | 2.10 (1.50, 69.7) |  | 3.09 (1.90, 221) |  | 4.74 (4.00, 182) | |  | | NP (0.63, 17) |  | NP (ND, 64) |  | 1.0 (0.57, 76) |  | 1.3 (0.71, 180) |  | 1.1 (0.54, 69) |  | NP (1.3, 140) | |  | 1.7 (NP, 99.4) |  | 2.0 (NP, 94.9) |  | NP, (2.1, NP) | |  | 0.55 (ND, 31.6) |  | ND (ND, 5.71) |  | 0.75 (ND,  22) | |  | NP (2.2, 41.9) | |  |
| BuP |  | 4.22 (4.49, 14.0) |  | 4.55  (4.76, 13.6) |  | 6.49  (6.75, 14.6) |  | 5.67  (5.92, 14.3) |  | 5.41  (5.85, 14.2) |  | NP (ND, 0.30) |  | NP (ND, 1.50) |  | NP (ND, 5.00) | |  | | NP (ND, 17) |  | NP (ND, 0.32) |  | NP (ND, 0.73) |  | NP (ND, 0.93) |  | NP (ND, 1.7) |  | NP (ND, 5.2) | |  | NP (NP, NP) |  | NP (NP, NP) |  | NP (NP, NP) | |  | NP (NP, NP) |  | NP (NP, NP) |  | NP  (NP, NP) | |  | NP (ND, 10.3) | |  |
| GM (median, P95)  ^a^ This study  ^b^ NHANES: Fourth National Report on Human Exposure to Environmental Chemicals Updated Tables, March 2021. Volume Two: NHANES 2011-2016 (2013-2014)  ^c^ CHMS: Results of the Canadian Health Measures Survey Cycle 5 (2012-2013)  ^d^ Hong et al., 2021  ^e^ Lee et al., 2021  ^f^ Murawski et al., 2021.  ^g^ Moos et al., 2015 | | | | | | | | | | | | | | | | | | | | | | | | | | | | | | | | | | | | | | | | | | | | | | | |  |  |

**Table S3. Paraben daily intake DI (μg/kg bw/day), hazard quotient (HQ) and hazard index (HI) of all population, minors and adults.**

|  | All (n= 1967) | | | | |  | Minors (n=622) | | | | |  | Adults (n=1345) | | | | |
| --- | --- | --- | --- | --- | --- | --- | --- | --- | --- | --- | --- | --- | --- | --- | --- | --- | --- |
|  | MeP | EtP | PrP | BuP | HI |  | MeP | EtP | PrP | BuP | HI |  | MeP | EtP | PrP | BuP | HI |
| DI - P50 | 38.7 | 4.89 | 20.8 | 1.71 | – |  | 40.0 | 4.94 | 20.6 | 1.65 | – |  | 39.4 | 4.86 | 20.9 | 1.73 | – |
| DI - P95 | 151 | 20.4 | 74.9 | 7.24 | – |  | 153 | 21.1 | 75.9 | 6.83 | – |  | 149 | 20.2 | 74.1 | 7.35 | – |
| DI - MAX | 1295 | 82.8 | 571 | 43.8 | – |  | 646 | 82.8 | 332 | 26.8 | – |  | 1295 | 76.5 | 571 | 43.8 | – |
| based on ADI by EFSA (2004) and thresholds derived by Moos et al. (2017) | | | | | | | | | | | | | | | | | |
| HQ/HI – P50 | 0.004^a^ |  | 1.04 | 0.085 | 1.14 |  | 0.004 ^a^ |  | 1.03 | 0.082 | 1.11 |  | 0.005 ^a^ |  | 1.05 | 0.086 | 1.14 |
| HQ/HI – P95 | 0.017 ^a^ |  | 3.74 | 0.36 | 4.07 |  | 0.017 ^a^ |  | 3.80 | 0.341 | 4.19 |  | 0.017 ^a^ |  | 3.71 | 0.368 | 4.05 |
| HQ/HI - MAX | 0.137 ^a^ |  | 28.6 | 2.19 | 30.9 |  | 0.072 ^a^ |  | 16.6 | 1.34 | 18.0 |  | 0.137 ^a^ |  | 28.5 | 2.19 | 30.9 |
| n > 1 (%) | 0 |  | 51.8 | 0.31 | 55.4 |  | 0 |  | 43.1 | 0.16 | 47.1 |  | 0 |  | 55.8 | 0.37 | 59.2 |

^a^ The sum of MeP and EtP

N > 1 (%) = percentage of samples with HQ/HI > 1.


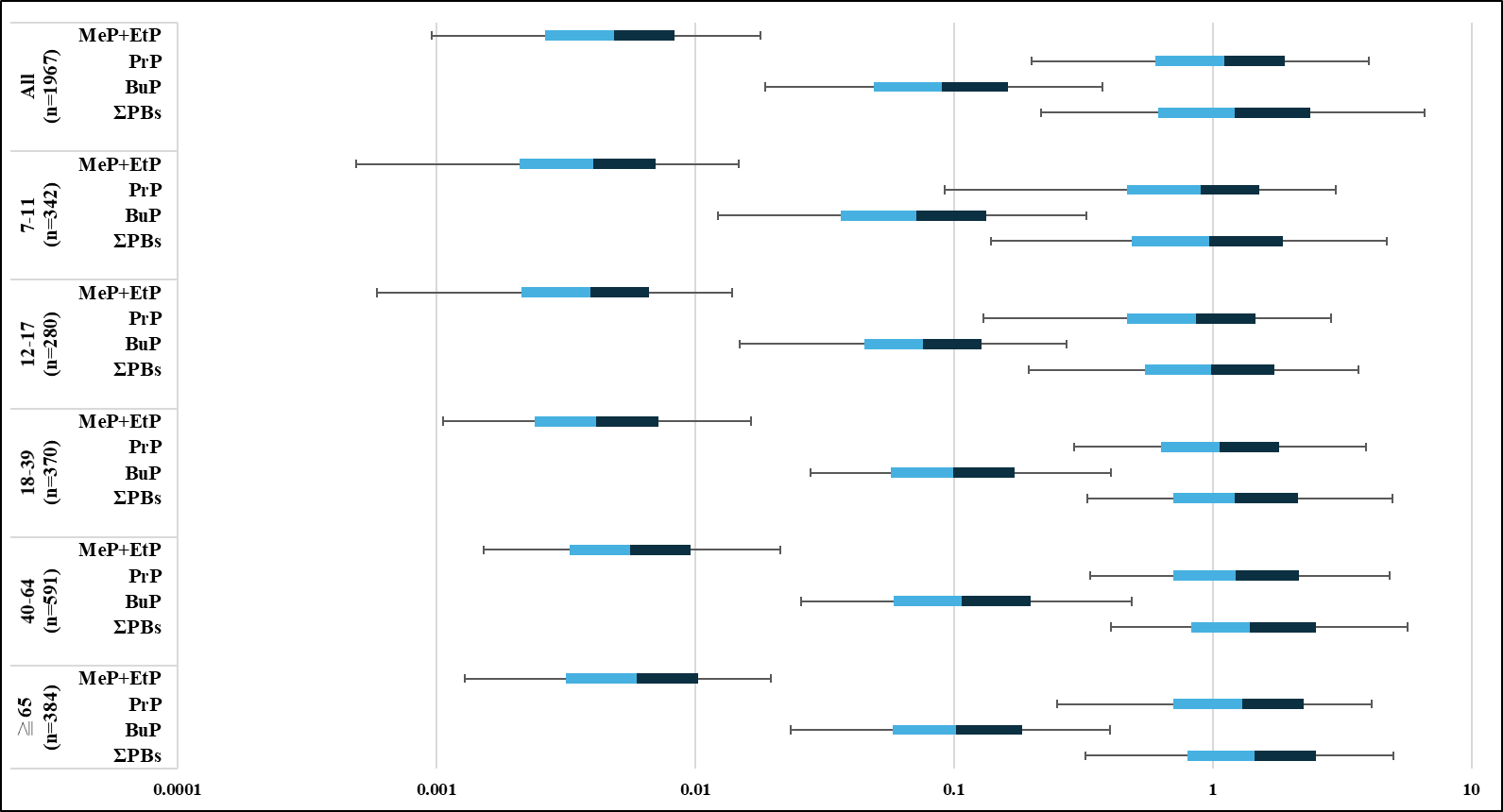


**Fig S1. The hazard quotient of general Taiwanese by different age group.**


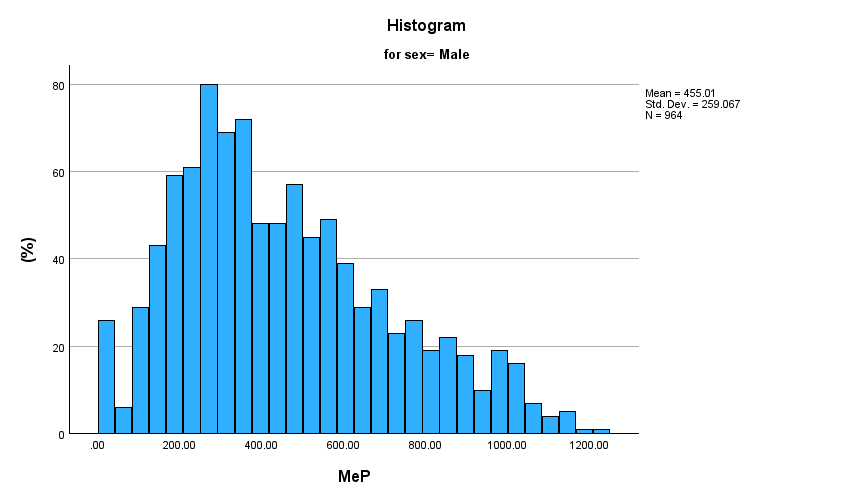

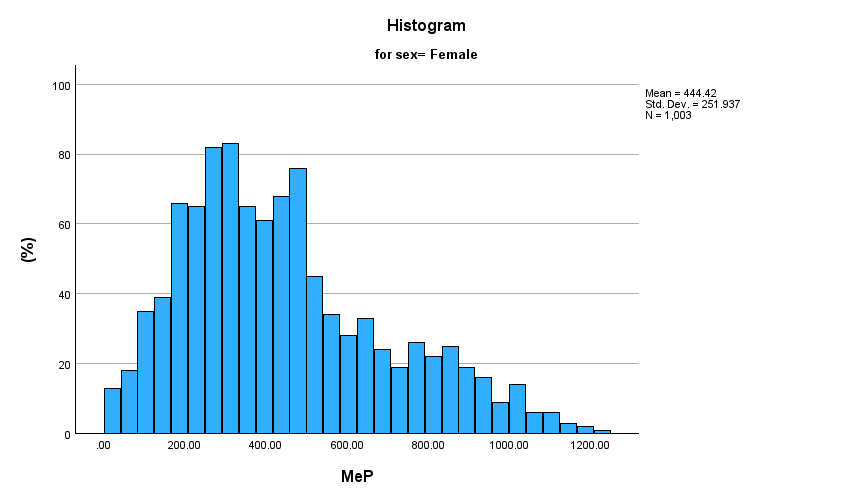

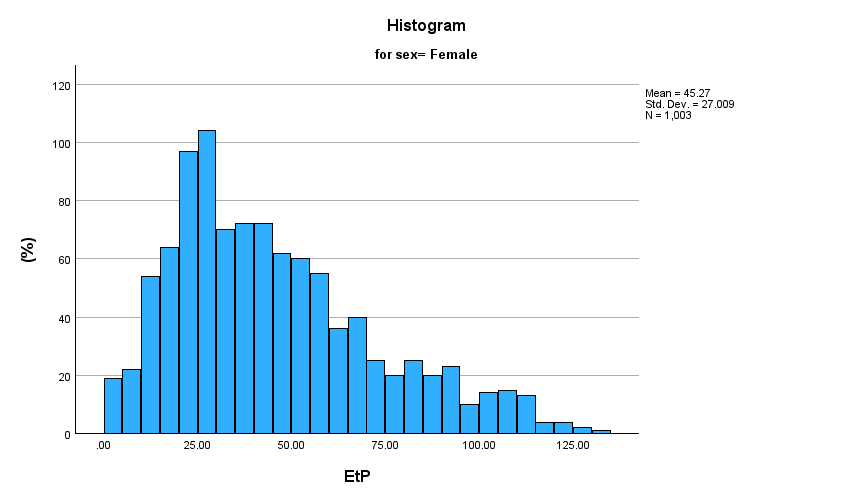

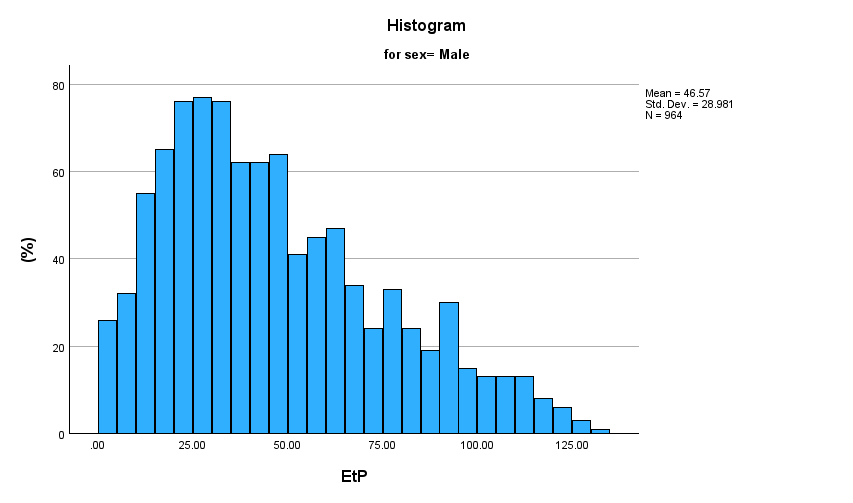


**Fig S2. Distribution of**

**Fig S2. Distribution of urinary paraben levels in Taiwanese among different sex.**


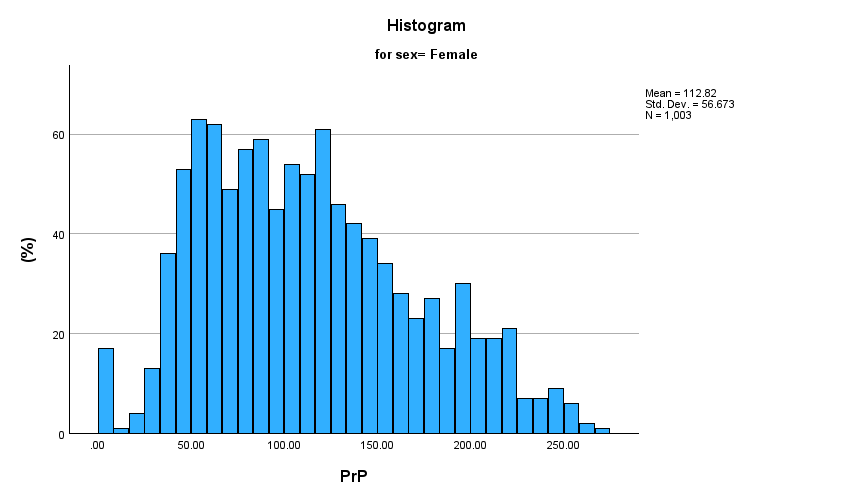

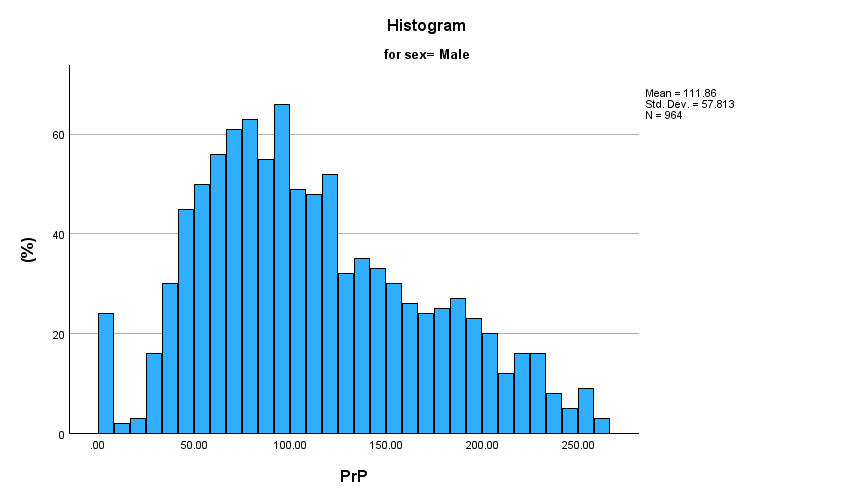

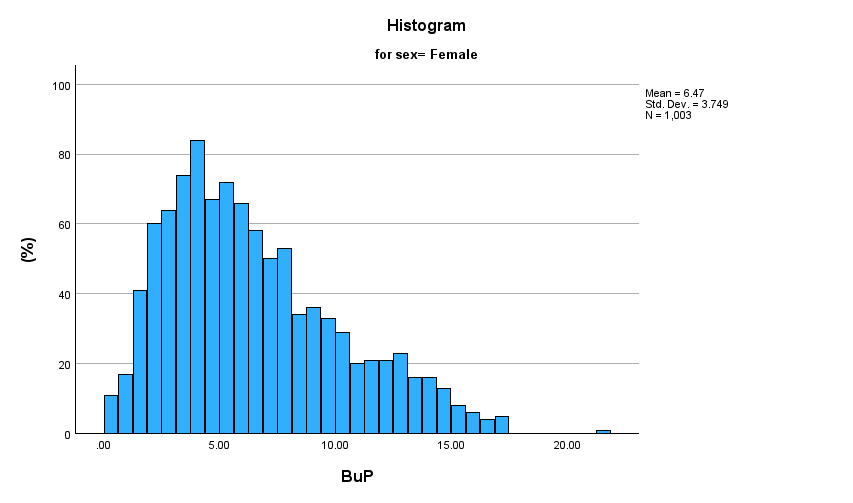

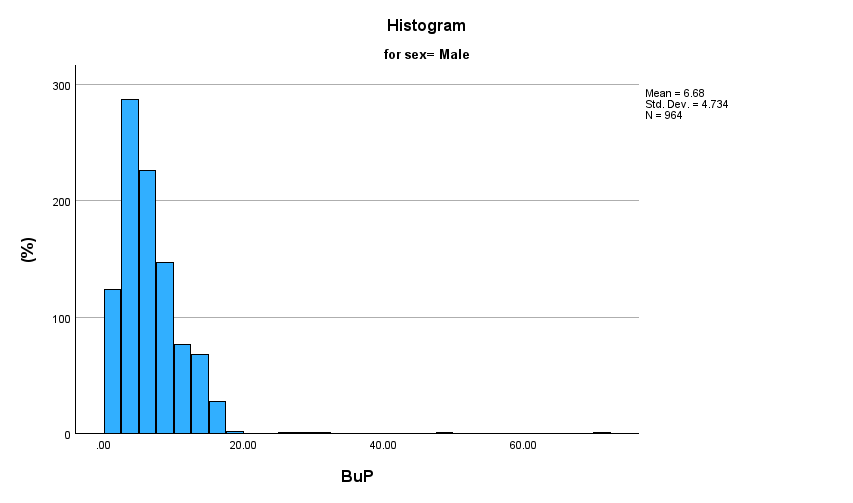

Supplement: Supplementary file 1 — Table S1: Urinary levels of parabens in Taiwanese among different residential area. Table S2: Comparison of urinary level of parabens (μg/L) in general population among different countries. Table S3: Paraben daily intake DI (μg/kg bw/day), hazard quotient (HQ) and hazard index (HI) of all population, minors and adults. Figure S1: The hazard quotient of general Taiwanese by different age group. Figure S2: Distribution of urinary paraben levels in Taiwanese among different sex. [file KJM2-9999-e70208-s001.docx]
